# Professor Ricord

**Published:** 1889-11

**Authors:** 


### Obituary.

Professor Ricord, of Paris, known generally as "the great Ricord," the father of modern syphilography, died in Paris, Oct. 22, 1889. He was an American by birth, his birthplace being Baltimore, Md.

